# Serum endocan levels in non-ST segment elevation myocardial infarction patients

**DOI:** 10.3389/fcvm.2026.1783106

**Published:** 2026-06-03

**Authors:** Adem Melekoglu, Alp Giray Aydin, Uğur Kahveci, Nazmiye Ozcan, Serkan Ceritli, Zeynep Mine Yalcinkaya Kara, Hakan Kilci, Ertugrul Altinbilek, Derya Ozturk

**Affiliations:** 1 Sisli Hamidiye Etfal Education and Research Hospital, Emergency Medicine, İstanbul, Türkiye; 2 Department of Emergency Medicine, Ministry of Health Eskişehir City Hospital, Eskişehir, Turkiye; 3 Emergency Department, University of Health Sciences Gulhane Training and Research Hospital, Ankara, Turkiye; 4 Department of Biochemistry, Sisli Hamidiye Etfal Education and Research Hospital, Istanbul, Turkiye; 5 Department of Cardiology, Sisli Hamidiye Etfal Education and Research Hospital, Istanbul, Turkiye

**Keywords:** coronary stenosis, endocan, endothelial dysfunction, inflammation, NSTEMI

## Abstract

**Background:**

Endothelial dysfunction and inflammation play a key role in the pathophysiology of acute coronary syndrome (ACS). Endocan has emerged as a novel biomarker reflecting endothelial activation.

**Objective:**

To investigate serum endocan levels in patients with non–ST-segment elevation myocardial infarction (NSTEMI) and their association with coronary stenosis severity.

**Methods:**

This prospective observational study included 81 NSTEMI patients undergoing coronary angiography. Patients were classified into clinically significant coronary stenosis and non-significant coronary stenosis groups. Serum endocan levels were measured at admission.

**Results:**

Patients who underwent coronary angiography (*n* = 81) were divided into two groups as those with clinically significant coronary stenosis (*n* = 40) and non-significant coronary stenosis (*n* = 41). Serum endocan levels were significantly higher in the group with clinically significant coronary stenosis (*p* < 0.001). Mean age and body mass index (BMI) were higher and statistically significant in the group with clinically significant coronary stenosis (*p* = 0.038, *p* = 0.042). Inflammation parameters C-reactive protein (CRP), White Blood Cell (WBC) and Neutrophil Lymphocyte ratio (NLR) were higher in the group with clinically significant coronary stenosis (*p* < 0.001, *p* = 0.011, *p* = 0.038). In multivariate logistic regression analysis, endocan (*p* < 0.001, 95% CI: 1.010–1.035), BMI (*p* = 0.020, 95% CI: 1.070–2.237), and CRP (*p* = 0.039, 95% CI: 1.003–1.099) were independently associated with clinically significant coronary stenosis. In Receiver operating curve (ROC) analysis performed to discriminate between patients with and without clinically significant coronary stenosis, the endocan cut-off value was determined as > 393.1 ng/L [*p* < 0.001, The Area Under the Curve (AUC) = 0.826, 95% CI = 0.732–0.919, Likelihood Ratio + = 7.65, LR - = 0.28].

**Conclusion:**

Endocan levels are elevated in NSTEMI patients with clinically significant coronary stenosis and may reflect endothelial dysfunction and atherosclerotic burden.

## Highlights

- Serum endocan levels are significantly higher in NSTEMI patients with clinically significant coronary stenosis.
- Endocan is independently associated with angiographic severity but does not reflect myocardial injury.
- High-sensitivity troponin levels do not discriminate between critical and non-significant coronary stenosis.
- Endocan showed good discriminatory ability to distinguish between patients with and without clinically significant coronary stenosis (AUC = 0.826, 95% CI: 0.732–0.919).
- Systemic factors such as age, diabetes, and smoking may contribute to elevated endocan levels.

## Introduction

Every year, millions of patients present to the emergency department with chest pain. The challenge for emergency physicians is to differentiate patients with chest pain from emergency or non-emergency conditions such as acute coronary syndrome. To accurately diagnose these patients, physicians rely on a number of tools, including features in the history and physical examination, the patient's risk factors for coronary artery disease, electrocardiography (ECG), biomarkers, risk stratification scoring systems, and occasionally clinical data (1).

Coronary artery disease (CAD) refers to disease caused by partial or complete blockage of the coronary artery supplying the myocardium. Atherosclerosis is the most common cause of abnormal reduction of cardiac blood flow. Atherosclerosis is the primary cause of the majority of cardiovascular diseases (CVD). Atherosclerosis triggers an immune-inflammatory process and plays an important role in plaque development and destabilisation. Endothelial cell dysfunction is the primary complication in atherosclerotic lesions. The main clinical presentation of atherosclerosis is acute coronary syndrome (ACS), which refers to a range of conditions ranging from unstable angina, non-ST segment elevation myocardial infarction (NSTEMI), to ST segment elevation myocardial infarction (STEMI). Acute coronary syndrome usually occurs after atherosclerotic plaque rupture and partial or complete occlusion of a vessel lumen (2).

Endothelial dysfunction is considered an early change in atherogenesis. Increased levels of systemic inflammatory markers are associated with cardiovascular disease (CVD). Endocan (endothelial cell-specific molecule-1) is a potential immunoinflammatory marker that may be linked to CVD. Endocan is released by vascular endothelial cells in various organs. Endocan may play an important role in the regulation of cell adhesion and elevated plasma levels may reflect endothelial dysfunction (3). Vascular endothelial dysfunction is the main factor in the progression of atherosclerosis. Endocan stimulates endo thelial cells to produce more types of inflammatory cytokines, increase vascular permeability and promote leukocyte migration, which plays an important role in the pathogenesis of various stages of atherosclerosis. Endocan, a novel endothelial mediator, regulates vascular smooth muscle cell migration and proliferation and thus may lead to the occurrence of atherosclerosis (4).

Unlike segment elevation on ECG, which is a rapid diagnostic tool in STEMI, clinical status, troponin and risk stratification are used for diagnosis in NSTEMI patients. Biomarkers have emerged as the main endpoints for the diagnosis, risk stratification and prognosis assessment of ACS (5). Among these biomarkers, troponin, especially high-sensitivity troponin (hs-cTn), has revolutionised the diagnosis of ACS with its superior sensitivity and negative predictive value (6). However, challenges such as specificity, standardisation and interpretation remain. Beyond troponins, a number of biomarkers reflecting myocardial damage, neurohormonal activation, inflammation, thrombosis and other pathways are being investigated to improve ACS management (7).

Since atherosclerosis-related endothelial dysfunction and inflammation processes are effective in NSTEMI patients, we aimed to investigate serum endocan levels in these patients.

## Methods

### Type and ethical aspects of the research

Our study was a single-center, prospective, observational study investigating cardiac biomarkers and serum endocan levels in patients who presented to the emergency department of our hospital and were diagnosed with NSTEMI and were found to have clinically significant coronary stenosis and non-significant coronary stenosis after coronary angiography. Ethical approval for the study was obtained from the Clinical Research Ethics Committee of the Health Sciences University Şişli Hamidiye Etfal Training and Research Hospital Health Practices and Research Center, with decision number 4,170 dated 21/11/2023. All eligible patients were informed about the study and their written consent was obtained. Patients who did not give written consent were excluded from the study.

### Study population and data collection

**Figure 1:**
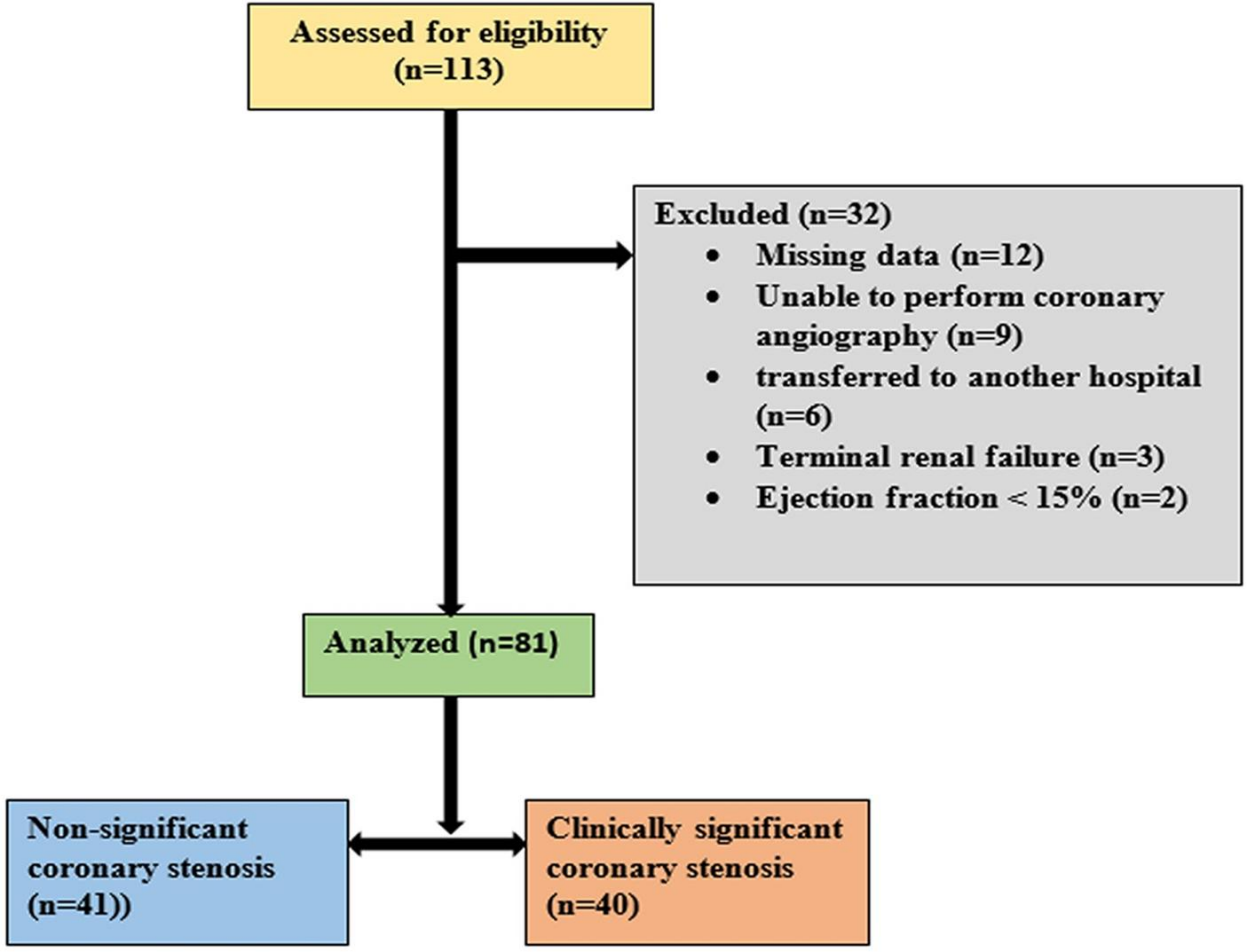
Flow diagram of the study.

Patients presenting to the emergency department with symptoms suggestive of acute coronary syndrome, such as chest pain and dyspnea, and subsequently diagnosed with non–ST-segment elevation myocardial infarction (NSTEMI) were consecutively and prospectively enrolled between December 1, 2023, and March 1, 2024. NSTEMI was defined according to the criteria of the European Society of Cardiology, based on the presence of ischemic symptoms in combination with a rise and/or fall in cardiac troponin levels without persistent ST-segment elevation on electrocardiography. At the time of admission (0 h), blood samples were obtained for routine hematological parameters and serum endocan levels. Additional blood samples for high-sensitivity cardiac troponin (hs-cTnI) were collected at presentation (0 h = hs-cTnI 1) and at 1 h (=hs-cTnI 2), in accordance with the rapid diagnostic protocol. Endocan levels were measured using a standardized enzyme-linked immunosorbent assay (ELISA) method according to the manufacturer's instructions. Laboratory analyses were performed by personnel blinded to the clinical and angiographic data. Risk stratification was performed using the Global Registry of Acute Coronary Events (GRACE) score, calculated at admission based on established clinical and laboratory parameters (8). Patient Information Management System (PANATES) was used for data recording. Demographic characteristics of the patients including age, gender, height, weight, vital parameters including systolic blood pressure, diastolic blood pressure, pulse rate, respiratory rate, oxygen saturation, and history of previous or known chronic diseases were recorded. The hemogram, biochemistry, and blood gas results of the patients were obtained from this record system. A total of 113 patients were initially evaluated for eligibility. The study flow diagram, including inclusion and exclusion processes and the final study population, is presented in Figure 1.

### Laboratory measurements

Venous blood samples were collected from the antecubital vein into serum separator tubes (yellow cap gel tubes; Sarstedt, Germany). Samples were allowed to clot at room temperature for 20 min and were then centrifuged at 3,000 rpm for 20 min. The separated serum was aliquoted and stored at −80 °C until collective analysis. Serum endocan levels were measured using a commercially available micro–enzyme-linked immunosorbent assay (ELISA) kit [Human Endothelial Cell-Specific Molecule-1 (Endocan) ELISA Kit; Bioassay Technology Laboratory, China], in accordance with the manufacturer's instructions. Calibration standards of known concentrations were prepared using the standard solutions provided in the kit. Optical density values were measured at 450 nm using a microplate reader (ELx800 Absorbance Microplate Reader; BioTek Instruments, USA). Endocan concentrations were calculated from the standard calibration curve and expressed in ng/L. The analytical measurement range of the assay was 5–2000ng/L. All serum samples were analyzed in a single batch to minimize inter-assay variability.

### Angiographic severity-based grouping

Patients were classified into two groups according to coronary angiography findings: clinically significant coronary stenosis and non-significant coronary stenosis. Clinically significant coronary stenosis was defined as the presence of: ≥70% luminal narrowing in at least one major epicardial coronary artery, or ≥50% stenosis in the left main coronary artery. Lesions with <50% stenosis were considered non-significant coronary stenosis. Intermediate lesions (50%–70% stenosis) were further evaluated using fractional flow reserve (FFR), when available. Lesions with FFR ≤0.80 were considered functionally significant and classified as clinically significant coronary stenosis, whereas those with FFR >0.80 were categorized as non-significant coronary stenosis. Thus, final group allocation was primarily based on anatomical severity, with functional assessment applied selectively in intermediate lesions. Coronary stenosis severity was visually assessed by experienced interventional cardiologists. Visual assessment was performed in accordance with current guideline-recommended angiographic thresholds, although interobserver variability cannot be excluded. Quantitative coronary angiography (QCA) was not routinely performed (9–11). Although the extent of coronary artery disease (single- vs. multivessel involvement) was documented, it was not included in the primary analysis, as the study was primarily designed to evaluate the relationship between endocan levels and angiographic severity rather than disease burden.

### Bias

Several sources of bias should be considered. First, as a single-center study conducted in a tertiary referral hospital, selection bias may be present, with a higher likelihood of enrolling patients with more severe disease and comorbidities. Second, verification bias may have occurred, as only patients undergoing coronary angiography were included in the final analysis. Third, residual confounding cannot be excluded, particularly due to the influence of systemic inflammatory and metabolic conditions such as diabetes mellitus and advanced age on endocan levels.

### Statistical analysis

An *a priori* sample size calculation was performed using G*Power software (version 3.1.9.7). Assuming a two-tailed alpha level of 0.05, statistical power (1–*β*) of 0.80, and a large effect size (Cohen's d = 0.8), the minimum required sample size was calculated as 52 patients (26 per group). The final study population (*n* = 81) exceeded this requirement, indicating that the study was adequately powered to detect a significant difference between groups. Continuous variables were expressed as mean ± standard deviation or median (interquartile range), and categorical variables as frequencies and percentages. Normality was assessed using the Kolmogorov–Smirnov test. Between-group comparisons were performed using the independent samples t-test or Mann–Whitney U test, as appropriate, and categorical variables were compared using the chi-square or Fisher's exact test. Correlations were evaluated using Spearman's test. Factors associated with clinically significant coronary stenosis were analyzed using univariate and multivariate logistic regression, and results were reported as odds ratios with 95% confidence intervals. Variables with *p* < 0.10 in univariate analysis and clinically relevant variables were included in the multivariate model. Receiver operating characteristic (ROC) curve analysis was performed to assess the discriminatory ability of endocan levels. A two-sided *p*-value <0.05 was considered statistically significant. All analyses were conducted using IBM SPSS Statistics software (version 28.0, Armonk, NY, USA).

## Results

The study included 81 patients. According to coronary angiography, they were divided into two groups as clinically significant coronary stenosis (*n* = 40) and non-significant coronary stenosis (*n* = 41). Major adverse cardiac event (MACE) developed in 2 patients in the clinically significant coronary stenosis patient group. No mortality was observed in any patient within 1 month (not shown in the table). Table 1 shows that patients with clinically significant coronary stenosis were significantly older (60.1 ± 11.4 vs. 54.9 ± 10.7 years, *p* = 0.038), had higher body mass index (*p* = 0.042) and GRACE score (*p* = 0.031) compared to those without clinically significant coronary stenosis, while all other baseline characteristics, including vital signs and comorbidities, were similar between groups (*p* > 0.05).

**Table 1:** Baseline characteristics of patients With and without clinically significant coronary stenosis.

Variable	Unit	Non-significant stenosis (*n* = 41)	Significant stenosis (*n* = 40)	*p*-value
Age	years	54.9 ± 10.7	60.1 ± 11.4	0.038
Body mass index	kg/m^2^	26.17 ± 1.8	27.1 ± 2.0	0.042
Systolic BP	mmHg	124.7 ± 12.8	126.9 ± 20.3	0.546
Diastolic BP	mmHg	78 (75–80)	80 (75–81)	0.238
Pulse	/min	78 (75–85)	78 (74–88)	0.906
Respiratory rate	/min	13 (12–15)	13 (12–15)	0.181
Oxygen saturation	%	97 (96–98)	96 (95–98)	0.529
Chest pain duration	min	240 (150–540)	180 (120–330)	0.207
Ejection fraction	%	55 (55–60)	55 (50–60)	0.163
GRACE score	—	124.5 ± 16.2	133.9 ± 21.7	0.031
Gender (male)	*n* (%)	32 (78.0)	31 (77.5)	0.953
Smoking	*n* (%)	8 (19.5)	13 (32.5)	0.280
CAD	*n* (%)	14 (34.1)	12 (30.0)	0.872
HT	*n* (%)	12 (29.3)	17 (42.5)	0.312
DM	*n* (%)	7 (17.1)	12 (30.0)	0.267
CHF	*n* (%)	4 (9.8)	3 (7.5)	0.718
CVD	*n* (%)	4 (9.8)	4 (9.9)	0.971
HL	*n* (%)	8 (19.5)	9 (22.5)	0.741
CRF	*n* (%)	5 (12.2)	2 (5.0)	0.449
COPD	*n* (%)	6 (14.6)	5 (13.6)	0.779
Malignancy	*n* (%)	3 (7.3)	4 (10.0)	0.973

Values are presented as mean ± standard deviation or median (interquartile range) for continuous variables, and number (percentage) for categorical variables. Independent samples t-test, Mann–Whitney U test, and chi-square test were used as appropriate.

BP, blood pressure; CAD, coronary artery disease; HT, hypertension; DM, diabetes mellitus; CHF, congestive heart failure; CVD, cerebrovascular disease; HL, hyperlipidemia; CRF, chronic renal failure; COPD, chronic obstructive pulmonary disease; GRACE, Global Registry of Acute Coronary Events.

Table 2 shows that patients with clinically significant coronary stenosis had significantly higher levels of endocan (463.5 ± 156.1 vs. 328.8 ± 69.9 ng/L, *p* < 0.001), CRP (*p* < 0.001), WBC (*p* = 0.011), neutrophils (*p* = 0.021), NLR (*p* = 0.038), and TG/HDL ratio (*p* = 0.042), while HDL levels were significantly lower (*p* < 0.001); all other laboratory parameters, including hs-cTnI levels, were similar between groups (*p* > 0.05).

**Table 2:** Laboratory parameters comparison between groups.

Variable	Unit	Non-significant stenosis (*n* = 41)	Significant stenosis (*n* = 40)	*p*-value
Endocan	ng/L	328.8 ± 69.9	463.5 ± 156.1	<0.001
Glucose	mg/L	121 (102–145)	110 (104–125)	0.323
Creatinine	mg/dL	0.96 ± 0.27	0.91 ± 0.18	0.304
AST	U/L	23 (18–135)	27 (21–39)	0.156
C-reactive protein	mg/L	3.6 (1.8–12)	13 (6–24)	<0.001
pH	-	7.39 ± 0.05	7.40 ± 0.08	0.566
Lactate	mmol/L	2.2 ± 0.7	2.2 ± 1.3	0.949
Hemoglobin	g/L	13.9 ± 1.8	13.5 ± 1.8	0.386
Platelet	10^9/L	232 ± 63	251 ± 73	0.199
WBC	10^9/L	9.8 ± 3.4	11.9 ± 3.7	0.011
Neutrophil	10^9/L	6.4 ± 3.1	8.4 ± 4.2	0.021
Lymphocyte	10^9/L	2.2 ± 1.0	1.9 ± 0.5	0.228
NLR	-	2.69 (2.1–3.9)	3.40 (2.7–5.8)	0.038
LDL	mg/dL	115 ± 23	109 ± 24	0.265
HDL	mg/dL	51 ± 12	42 ± 9	<0.001
Triglyceride	mg/dL	134 ± 44	142 ± 57	0.501
TG/HDL	-	2.89 ± 1.5	3.67 ± 1.9	0.042
hs-cTnI 1	ng/mL	19 (11–59)	25 (7–117)	0.514
hs-cTnI 2	ng/mL	160 (55–416)	188 (53–734)	0.561

Values are presented as mean ± standard deviation or median (interquartile range). Independent samples t-test or Mann–Whitney U test was used as appropriate.

AST, aspartate aminotransferase; WBC, white blood cell; NLR, neutrophil-lymphocyte ratio; LDL, low-density lipoprotein; HDL, high-density lipoprotein; TG/HDL, triglyceride/HDL ratio; hs-cTnI, high-sensitivity cardiac troponin I.

Table 3 shows that in univariate analysis, endocan (OR = 1.019, *p* < 0.001), BMI (OR = 1.275, *p* = 0.047), age (OR = 1.044, *p* = 0.043), CRP (OR = 1.032, *p* = 0.034), WBC (OR = 1.176, *p* = 0.015), HDL (OR = 0.930, *p* = 0.001), hs-cTnI 1 (OR = 1.007, *p* = 0.033), and hs-cTnI 2 (OR = 1.001, *p* = 0.040) were significantly associated with clinically significant coronary stenosis, whereas in multivariate analysis only endocan (OR = 1.022, 95% CI: 1.010–1.035, *p* < 0.001), BMI (OR = 1.547, 95% CI: 1.070–2.237, *p* = 0.020), and CRP (OR = 1.050, 95% CI: 1.003–1.099, *p* = 0.039) remained independent predictors, while other variables lost statistical significance (*p* > 0.05).

**Table 3:** Logistic regression analysis of factors associated with clinically significant coronary stenosis.

Variable	Univariate OR	95% CI	*p*-value	Multivariate OR	95% CI	*p*-value
Endocan	1.019	1.009–1.028	<0.001	1.022	1.010–1.035	<0.001
BMI	1.275	1.003–1.619	0.047	1.547	1.070–2.237	0.020
Age	1.044	1.001–1.089	0.043	0.997	0.927–1.072	0.925
Ejection fraction	0.937	0.873–1.005	0.068	1.031	0.914–1.163	0.616
CRP	1.032	1.002–1.063	0.034	1.050	1.003–1.099	0.039
WBC	1.176	1.032–1.340	0.015	1.210	0.974–1.504	0.085
HDL	0.930	0.891–0.971	0.001	0.938	0.875–1.007	0.076
hs-cTnI 1	1.007	1.001–1.013	0.033	1.000	0.987–1.014	0.954
hs-cTnI 2	1.001	1.001–1.002	0.040	1.000	1.000–1.001	0.486
Constant			0.000			0.006

BMI, body mass index; WBC, white blood cell; HDL, high-density lipoprotein; CRP, C-reactive protein; hs-cTnI, high-sensitivity cardiac troponin I.

All variance inflation factor (VIF) values were below 2.00, indicating no evidence of significant multicollinearity among the independent variables.

The Hosmer–Lemeshow test indicated good model fit (*χ*^2^ = 3.302, p = 0.914).

Table 4 shows the correlation analysis between Endocan levels and other independent variables. Endocan levels correlated moderately-weakly with age, Hs-cTnI1, weakly positively with WBC, CRP (r = 0.417, r = 0.278, r = 0.232, r = 0.352, respectively). It showed a modest negative correlation with ejection fraction and a weak negative correlation with HDL (r = −0.430, r = −0.330, respectively).

**Table 4:** Correlation analysis of Serum endocan levels with independent variables.

Variable	Correlation coefficient (r)	*p*-value
Age	0.417	<0.001
BMI	0.076	0.498
Ejection fraction	−0.430	<0.001
HDL	−0.330	<0.001
WBC	0.278	0.012
CRP	0.232	0.037
hs-cTnI 1	0.352	0.001
hs-cTnI 2	0.098	0.383

Values represent Spearman correlation coefficients (r).

BMI, body mass index; HDL, high-density lipoprotein; WBC, white blood cell; CRP, C-reactive protein; hs-cTnI, high-sensitivity cardiac troponin I.

**Figure 2:**
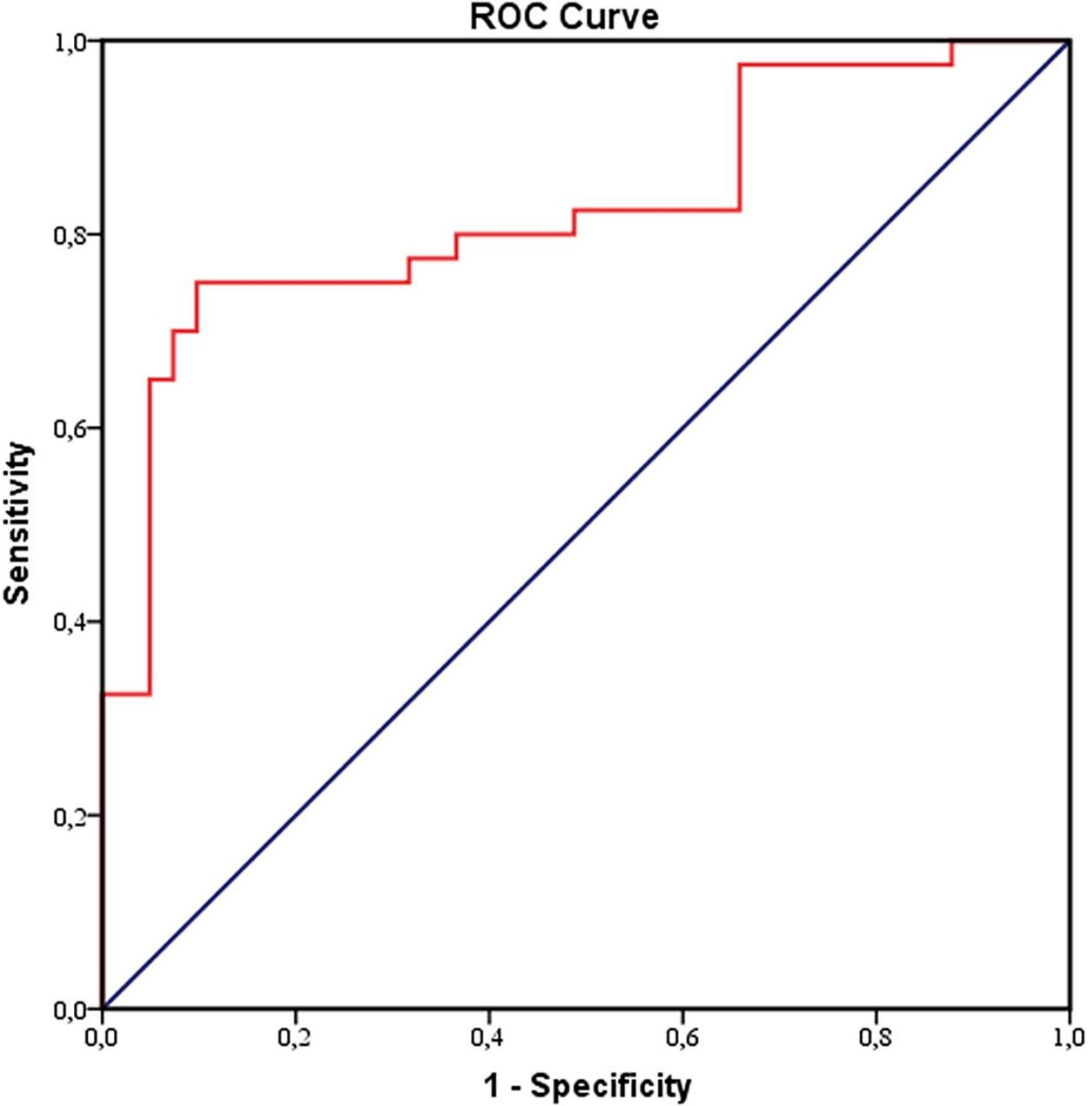
ROC analysis of endocan for clinically significant coronary stenosis.

Receiver operating characteristic (ROC) curve analysis was performed to evaluate the ability of endocan levels to discriminate between patients with and without clinically significant coronary stenosis. Endocan demonstrated good discriminatory performance for clinically significant coronary stenosis (AUC = 0.826, 95% CI: 0.732–0.919). The optimal cut-off value of endocan for discriminating clinically significant coronary stenosis was 393.1 ng/L. Sensitivity and specificity were 75.0% and 90.2%, respectively (Figure 2. ROC analysis of endocan levels for the clinically significant coronary stenosis group). Likelihood Ratio+was 7.65 and Likelihood Ratio - was 0.28 (not shown in the table).

Table 5 shows that patients with higher endocan levels (>393.1 ng/L) were significantly older (62.5 ± 11.5 vs. 53.8 ± 9.7 years, *p* < 0.001) and had lower HDL levels (*p* < 0.001), higher TG/HDL ratio (*p* = 0.001), higher CRP levels (*p* = 0.013), and higher COHb levels (*p* = 0.016); additionally, smoking (41.2% vs. 14.9%, *p* = 0.016) and diabetes mellitus (38.2% vs. 12.8%, *p* = 0.016) were more frequent in this group, while other clinical and laboratory parameters, including hs-cTnI levels, were similar between groups (*p* > 0.05).

**Table 5:** Comparison of clinical and laboratory characteristics by endocan Cut-off level.

Variable	Unit	Endocan < 393.1 (*n* = 47)	Endocan > 393.1 (*n* = 34)	*p*-value
Age	years	53.8 ± 9.7	62.5 ± 11.5	<0.001
Body mass index	kg/m^2^	26.5 ± 1.85	26.8 ± 2.1	0.543
Chest pain duration	min	180 (140–570)	180 (120–240)	0.220
Ejection fraction	%	55 (55–57)	55 (50–60)	0.277
hs-cTnI 1	ng/mL	19.0 (11–58)	30.0 (7.5–106)	0.358
hs-cTnI 2	ng/mL	160 (54–483)	192 (55–511)	0.660
LDL	mg/dL	117 ± 24	107 ± 22	0.065
HDL	mg/dL	51.2 ± 12.8	41.0 ± 9.2	<0.001
Triglyceride	mg/dL	130 ± 48	148 ± 53	0.123
TG/HDL	-	2.84 ± 1.6	3.88 ± 1.8	0.001
Creatinine	mg/dL	0.96 ± 0.25	0.89 ± 0.18	0.194
C-reactive protein	mg/L	4.3 (2.2–14.6)	11.4 (5.4–24.0)	0.013
WBC	10^9/L	10.4 ± 3.5	11.5 ± 3.9	0.192
Lactate	mmol/L	2.3 ± 1.2	2.1 ± 0.7	0.506
COHb	%	0.2 (0.05–0.85)	1.25 (0.1–4.5)	0.016
Gender (male)	*n* (%)	37 (78.7)	26 (76.5)	0.810
Smoking	*n* (%)	7 (14.9)	14 (41.2)	0.016
CAD	*n* (%)	13 (27.7)	13 (38.2)	0.314
HT	*n* (%)	14 (29.8)	15 (44.1)	0.184
DM	*n* (%)	6 (12.8)	13 (38.2)	0.016
CHF	*n* (%)	4 (8.5)	3 (8.8)	0.629
CVD	*n* (%)	5 (10.6)	3 (8.8)	0.549
HL	*n* (%)	7 (14.9)	19 (29.4)	0.191
CRF	*n* (%)	5 (10.6)	2 (5.8)	0.371
COPD	*n* (%)	8 (17)	3 (8.8)	0.463
Malignancy	*n* (%)	4 (8.5)	3 (8.8)	0.961

Values are presented as mean ± standard deviation or median (interquartile range) for continuous variables, and number (percentage) for categorical variables.

Independent samples t-test, Mann–Whitney U test, and chi-square test were used as appropriate.

CAD, coronary artery disease; HT, hypertension; DM, diabetes mellitus; CHF, congestive heart failure; CVD, cerebrovascular disease; HL, hyperlipidemia; CRF, chronic renal failure; COPD, chronic obstructive pulmonary disease; TG/HDL, triglyceride/HDL ratio; hs-cTnI, high-sensitivity cardiac troponin I.

## Discussion

In our study, we found that serum endocan levels were higher in patients with clinically significant coronary stenosis in coronary angiography performed with a diagnosis of NSTEMI compared to patients without clinically significant coronary stenosis. We found that endocan levels were moderately to weakly positively correlated with the Hs-cTnI1 level taken at the time of initial presentation to the emergency department. We observed that endocan levels were higher in elderly patients, patients with DM and smokers.

In recent meta-analyses and observational studies, circulating endocan levels have been shown to be significantly elevated in patients with acute coronary syndrome (ACS), reflecting endothelial activation and systemic inflammation rather than direct myocardial injury (12–14). According to the Fourth Universal Definition of Myocardial Infarction, both STEMI and NSTEMI require objective evidence of myocardial injury with necrosis, defined by a rise and/or fall in cardiac troponin values with at least one value above the 99th percentile. Instead, clinical scenarios may include unstable angina without myocardial necrosis or myocardial injury unrelated to ischemia, such as in heart failure or chronic kidney disease (15, 16). In the context of NSTEMI, elevated high-sensitivity troponin reflects myocardial injury, whereas increased endocan levels may provide complementary information by capturing underlying endothelial dysfunction and inflammatory activity.

Previous studies have reported a weak or inconsistent relationship between endocan and cardiac troponin levels in ACS, which may be explained by their distinct pathophysiological roles (13, 17). Cardiac troponins remain the gold standard biomarkers for myocardial necrosis due to their high specificity for cardiomyocyte injury; however, they do not adequately reflect endothelial dysfunction or atherosclerotic burden. In contrast, endocan is a marker of endothelial activation and vascular inflammation, which are central to the pathogenesis of atherosclerosis (12, 14). This mechanistic difference may explain the lack of strong correlation between troponin and endocan levels observed both in prior studies and in our findings.

Although cardiac troponin is widely used for diagnosis and risk stratification in ACS, its elevation is not specific to ischemic myocardial infarction and may occur in a variety of non-ischemic conditions, including heart failure, renal dysfunction, sepsis, and pulmonary embolism (15, 16). Therefore, the magnitude of troponin elevation alone should not be interpreted as a surrogate marker of coronary stenosis severity. In our study, hs-cTnI levels were elevated in both patients with and without clinically significant coronary stenosis, without a statistically significant difference between groups, indicating limited discriminatory value for anatomical severity. In contrast, endocan levels were significantly higher in patients with clinically significant coronary stenosis, suggesting an association with disease burden. However, this finding reflects an association rather than a validated predictive performance.

Endocan has also been shown to be elevated in several chronic systemic conditions associated with endothelial dysfunction, including hypertension, diabetes mellitus, cerebrovascular disease, and chronic kidney disease (18–20). In particular, patients with type 2 diabetes mellitus exhibit significantly higher circulating endocan levels, especially in the presence of poor glycemic control, likely due to chronic endothelial activation and low-grade inflammation (19, 20). In our cohort, the prevalence of type 2 diabetes mellitus was higher among patients with elevated endocan levels. This suggests that diabetes may act as a confounding factor contributing to increased endocan levels, rather than being solely attributable to coronary stenosis severity. Therefore, the observed association between elevated endocan levels and clinically significant coronary stenosis may be partially influenced by the higher burden of metabolic comorbidities.

Immunoinflammatory processes play a central role in the initiation and progression of cardiovascular diseases (CVDs), and circulating inflammatory cells have been identified as important predictors of disease occurrence and progression (21). In recent years, increasing attention has been directed toward low-grade inflammatory markers as contributors to cardiovascular risk and as potential tools for risk stratification (21). Patients with systemic inflammatory conditions have been shown to carry a substantially higher risk of developing CVD compared to the general population, further supporting the role of inflammation in atherogenesis (22). In the setting of acute coronary syndrome (ACS), pro-inflammatory cytokines such as interleukins, along with leukocyte subtypes, contribute to endothelial dysfunction and are widely used in clinical risk assessment models (22). Consistent with the literature, our findings demonstrated elevated inflammatory markers (including WBC, CRP, and NLR) in patients with NSTEMI. Notably, these markers were significantly higher in patients with clinically significant coronary stenosis, underscoring the contribution of systemic inflammation to disease severity in ACS.

Atherosclerosis is a chronic immunoinflammatory condition characterized by lipid accumulation within the vascular wall, leading to progressive luminal narrowing. Dyslipidemia parameters (including LDL, HDL, triglycerides, and the TG/HDL ratio) are well-established contributors to atherosclerosis and coronary artery disease (CAD) (23). However, the relationship between endocan and lipid parameters remains inconsistent across studies. Some reports have found no significant association between circulating endocan levels and lipid profiles (24), whereas others have demonstrated varying correlations, including a positive association with HDL or triglycerides and a negative relationship with HDL (25). In our study, the TG/HDL ratio was significantly higher in NSTEMI patients with clinically significant coronary stenosis, and a positive correlation was observed between endocan levels and the TG/HDL ratio. Additionally, endocan levels showed a negative correlation with HDL. These findings suggest that endocan may reflect an unfavorable lipid profile and support its potential role as a marker of atherosclerotic burden in NSTEMI.

### Limitations

This study has several limitations. First, the relatively small sample size may limit the generalizability of the findings. Second, coronary stenosis severity was assessed by visual estimation without routine use of quantitative or intravascular imaging techniques, which may have introduced measurement variability and reduced classification precision. Third, the absence of intracoronary imaging precluded detailed assessment of plaque morphology and vulnerability, limiting mechanistic interpretation. Finally, although adjustments were performed, unmeasured confounders related to metabolic and inflammatory status may have influenced endocan levels.

## Conclusion

Serum endocan levels are elevated in NSTEMI patients with clinically significant coronary stenosis and may reflect endothelial dysfunction and atherosclerotic burden. However, these findings indicate an association rather than a predictive capability. Systemic factors such as age, diabetes mellitus, and smoking may contribute to increased endocan levels. Further large-scale prospective studies are needed to clarify the clinical utility of endocan as a biomarker in NSTEMI.

## Data Availability

The raw data supporting the conclusions of this article will be made available by the authors, without undue reservation.
